# An Overview of Hazardous Impacts of Soil Salinity in Crops, Tolerance Mechanisms, and Amelioration through Selenium Supplementation

**DOI:** 10.3390/ijms21010148

**Published:** 2019-12-24

**Authors:** Muhammad Kamran, Aasma Parveen, Sunny Ahmar, Zaffar Malik, Sajid Hussain, Muhammad Sohaib Chattha, Muhammad Hamzah Saleem, Muhammad Adil, Parviz Heidari, Jen-Tsung Chen

**Affiliations:** 1 Key Laboratory of Arable Land Conservation, Ministry of Agriculture, College of Resources and Environment, Huazhong Agricultural University, Wuhan 430070, China; kamiagrarian763@gmail.com; 2 Department of Soil Science, University College of Agriculture and Environmental Sciences, The Islamia University of Bahawalpur, Bahawalpur 63100, Punjab, Pakistan; aasmaparveen452@gmail.com (A.P.); zafar_agrarian@yahoo.com (Z.M.); 3 College of Plant Science and Technology, Huazhong Agricultural University, Wuhan 430070, China; sohaibchattha08@gmail.com (M.S.C.); saleemhamza312@webmail.hzau.edu.cn (M.H.S.); 4 Stat Key Laboratory of Rice Biology, China National Rice Research Institute, Hangzhou 310006, China; hussainsajid@caas.cn; 5 College of Urban and Environmental Sciences, Northwest University, Xi’an 710127, China; adilmuhammad@stumail.nwu.edu.cn; 6 Department of Agronomy and Plant Breeding, Faculty of Agriculture, Shahrood University of Technology, Shahrood 3619995161, Iran; heidarip@shahroodut.ac.ir; 7 Department of Life Sciences, National University of Kaohsiung, Kaohsiung 811, Taiwan

**Keywords:** salinity, selenium (Se), crops, reactive oxygen species (ROS), enzymatic anti-oxidative system

## Abstract

Soil salinization is one of the major environmental stressors hampering the growth and yield of crops all over the world. A wide spectrum of physiological and biochemical alterations of plants are induced by salinity, which causes lowered water potential in the soil solution, ionic disequilibrium, specific ion effects, and a higher accumulation of reactive oxygen species (ROS). For many years, numerous investigations have been made into salinity stresses and attempts to minimize the losses of plant productivity, including the effects of phytohormones, osmoprotectants, antioxidants, polyamines, and trace elements. One of the protectants, selenium (Se), has been found to be effective in improving growth and inducing tolerance against excessive soil salinity. However, the in-depth mechanisms of Se-induced salinity tolerance are still unclear. This review refines the knowledge involved in Se-mediated improvements of plant growth when subjected to salinity and suggests future perspectives as well as several research limitations in this field.

## 1. Introduction

Various abiotic stresses, such as drought, heat, heavy metals, soil salinity, flooding, and cold, are responsible for the reduction of the growth, development, and productivity of crops worldwide [1]. Soil salinity is an overwhelming environmental threat to world food production and agricultural sustainability [2]. A soil with an electrical conductivity (EC) of saturated soil paste extract (ECe) in the plant root zone more significant than 4 dSm^−1^ (about 40 mM NaCl), 0.2 MPa osmotic stress [3] and an exchangeable sodium percentage (ESP) of 15% at 25 °C is termed as salt-affected soil [4]. Some of the most discussed reasons for soil salinity are poor soil-sustainable practices, excessive saline water irrigation and a severe usage of mineral fertilizers in arid and semi-arid regions (characterized by high evapotranspiration, high temperature, and low rainfall) across the globe [5]. The area under soil salinity is further enhanced by the conversion of fertile agricultural land into urban area, placing the efforts of scientists to produce 70% more food to feed the population of the world in 2050 of 9.3 billion at risk [6]. In 2001, almost 7% of the soils of the entire world were salt-affected in nature [7]. Globally, salinity is a significant abiotic stress, affecting one-quarter to one-third of the crop productivity of agricultural soils [8]. It was estimated in 2003 that up to the middle of the 21st century, the salinity-induced loss of cultivated soil will reach up to 50% [9]. In 2008, it was reported that, due to high salinization, 77 million hectares of the world’s total cultivated area (1.5 billion hectares) was adversely affected [10]. At present, about 10% of the global land area and 50% of irrigated areas are exposed to salinity, causing a loss of about 12 billion US$ in the agricultural sector [11].

**Figure 1:**
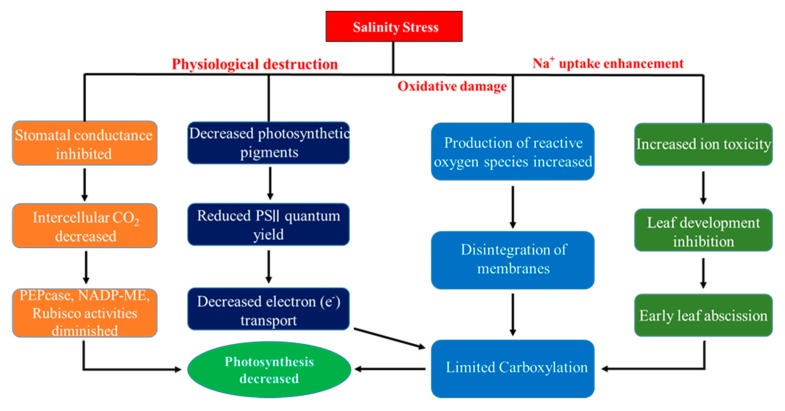
Schematic diagram interpreting the hazardous impacts of soil salinity stress in crop plants. The figure is briefly modified from the literature [20].

Soil salinity is a complex mechanism that is responsible for adverse effects on the physiological and biochemical pathways of crop plants [12]. Excess accumulation of Na^+^ induces efflux of cytosolic K^+^ and Ca^2+^, consequently, leading to imbalance in their cellular homeostasis, nutrient deficiency, oxidative stress, retarded growth, and cell death [13]. It has been reported in many previous studies that a high level of salinization drastically affects plant photosynthesis due to some stomatal restrictions; for example, stomatal closure [4] and/or non-stomatal restrictions comprising chlorophyll malfunctioning [14], deprivation of enzymatic proteins and membranes of photosynthetic apparatus [15], and chloroplast ultrastructure destruction [16]. Salt-affected soils have higher Na^+^/K^+^ and Na^+^/Ca^2+^ ratios because of the higher amount of Na^+^ in the soil solution. Hence, a reduction in K^+^ and Ca^+2^ uptakes cause the inhibition of the proper functioning of the cell, instability of cell membranes, and hindrance of enzymatic activities [17]. Moreover, some other secondary stresses, such as oxidative stress followed by osmotic pressure and ionic toxicity, are involved in the production of excessive reactive oxygen species (ROS) in cytosol, chloroplast, and mitochondria [2,4] such as O_2_^−^ (superoxide radicals), H_2_O_2_ (hydrogen peroxide), O_2_ (singlet oxygen) and OH^−^ (hydroxyl ions). These reactive oxygen species with strong oxidation ability can cause injuries to plant tissue, DNA mutation, cell membrane disruption [18], and the degradation of lipids, proteins, and photosynthetic pigments [19] (Figure 1).

The application of macro- and micro-nutrients is one of the management approaches for coping with environmental stresses such as soil salinity [21]. Selenium (Se) has been considered as a beneficial element for crop production which plays an important role in physio-biochemical processes [22,23]. Although higher plants do not require Se for their growth and development [24,25], supplementation of Se at lower dosages not only protects plants from ROS induced oxidative damage by activating the antioxidative mechanisms [22], but also improves the Se content in the edible parts of the crop plants [26]. Some studies have shown that Se is an essential element for human and animal, which plays some beneficial roles in higher plants. Selenium application caused an increasing growth in rice (*Oryza sativa* L.) [27] and wheat (*Triticum aestivum* L.) [28], under both stressed and non-stressed conditions. Se has been demonstrated to regulate plant growth by strengthening the stress tolerance mechanisms such as antioxidant and secondary metabolite metabolism [29]. It has also been reported previously that Se reversed the negative impacts of soil salinity on the photochemical efficiency of photosystem II [30]. Moreover, Se could also protect the metabolism and cellular functioning by up-regulating the ROS neutralizing pathways and the osmoregulatory mechanisms [28].

Although several excellent investigations have been done on Se induced salinity tolerance mechanisms in various crops, there is no comprehensive review on Se-mediated improvements in crops. In this review article, the role of Se in the improvement of common morpho-physiological and molecular responses of various crop plants subjected to salt stress are briefly discussed and some practical options have been proposed on how Se could play its role to induce salinity tolerance in crops.

## 2. Hazardous Impacts of Soil Salinity in Crops

Salinity stress is exceptional among all the abiotic stresses limiting crop yield efficiency in arid and semi-arid zones where natural conditions favor salinization due to insufficient precipitation for the leaching of salts [31]. According to the biphasic model of growth reduction via salinization [32], the detrimental impacts of salt-affected soils are coupled with a reduction of osmosis (primary phase) and ion cytotoxicity (secondary phase), in addition to the production of reactive oxygen species (ROS) and nutrient imbalance [4]. A high osmotic stress is linked with the accumulation of soluble salts in soil solution, leading to water stress due to a reduction in the stomatal aperture, which eventually hampers plant growth [33]. Ion cytotoxicity is the effect of the substitution of K^+^ and Ca^+2^ by Na^+^ and Cl^−^ in different biochemical reactions due to a higher salt concentration in the root zone of crop plants [34,35] (Figure 1).

### 2.1. Impacts of Salinity on Plant Agronomic Traits

Soil salinity is known for its adverse effects on plant growth and development [36]. However, the inhibitory effect of salt stress depends on various factors such as salt concentration, time interval, plant species and varieties, photochemical quenching capacity, plant growth stages, stress type, gas exchange characteristics, photosynthetic pigments, and environmental conditions [21]. It was concluded in various studies on *Zea mays* L. [37], *Oryza sativa* L. seedlings [38], *Vigna unguiculata* L. [39], *Brassica campestris* L. [40], and *Vicia faba* L. [41] that a low level of salinization increased plant length. However, higher concentrations of sodium chloride salt reduced the plant height of *Vigna mungo* L., [42], *Helianthus annuus* L. [43], and *Tanacetum parthenium* L. [44]. The increment in plant height was might be an effect of an adjustment of osmotic activity due to fewer soluble salts in the growth medium, while plant height reduction was an indication of adverse effects of excessive salts on the photosynthetic rate, a decreased level of carbohydrates and growth hormones (causing growth inhibition) and a reduction in protein synthesis by changing antioxidant enzyme activities [45].

Various studies revealed that the plant biomass (fresh and dry biomass), number of leaves and leaf area were drastically affected by salinity levels up to 8 dSm^−1^ [44,46,47]. In the context of plant growth, it has been reported by many researchers that dry matter production and plant growth retardation under salt-affected soils could be subjected to the inhibition of cell elongation [21] through the direct impairment of the activities of transport proteins such as H^+^-ATPase and H^+^-PPase [48]. Another reason for plant growth reduction could be the detrimental effects of salinity stress on photosynthesis, ultimately limiting plant and leaf growth and chlorophyll contents [49]. Furthermore, the fresh and dry biomass of *Brassica napus* L. cv. Talaye was significantly decreased, while root growth was less affected compared to shoot growth under salinity stress [47]. It was hypothesized that, under salinization, a low water uptake efficiency leads to lesser leaf area development than root growth, due to which soil moisture is conserved to prevent the accumulation of the vast amount of soluble salts in the soil [4,47]. Several studies have revealed that a high accumulation of Na^+^ and Cl^−^ ions in cell sap excites a low osmotic gradient in the nutrient medium, resulting in reduced water uptake, which in turn affects plant morphological characteristics [50]. It has been documented that high salt density is responsible for lower N accretion in plants due to the interaction between Cl^−^ and NO_3_^−^ and between Na^+^ and NH_4_^+^, which subsequently reduces plant growth and crop yield [51]. Another mechanism behind the reduction of plant growth under saline conditions might be the reduction in photosynthesis due to the plant stomatal closure and the resulting reduction of carbon uptake [21]. A significant reduction in the absorption of nutrient elements due to reduced osmotic pressure has also been reported as a secondary impact of salinity stress on reduced plant nourishment [52].

### 2.2. Impacts of Salinity on Physiological Traits

Soil salinization has been recognized as a severe threat to crop growth and yield, even in irrigated areas, worldwide [2]. It is estimated that salinity can reduce crop production in up to 20% of irrigated lands across the globe, and this loss will increase to about 50% of arable land up to mid-21st century [9]. Recently, various studies have reported that soil salinity stress causes reduction in the physiological attributes of cereal crops such as wheat (*Triticum aestivum* L.) [13,28] and mung bean (*Vigna radiata* L.) [53]. Plant growth and yield reduction induced by soil salinity might occur due to the changes in numerous physiological and biochemical attributes, i.e., the reduction of leaf chlorophyll content (Chl a, b, carotenoids) and photosynthesis capacity, as well as the alteration of energy in the mechanisms of ion exclusion, osmotic adjustment, and nutrient imbalance [54]. Mostly, salt-affected soils affect crops in three ways: osmotic stress, ion imbalance, and oxidative damage [55]. The main response of salt-affected soils is the toxic effects of sodium (Na^+^) and chloride (Cl^−^) ion accumulation in plant tissues [55,56]. It has been proven that plants under salinity stress accumulate more Na^+^ ions, resulting in the agitation of ionic balance and plant metabolism and stimulation of oxidative damage, while the K^+^ ion status in plant tissues helps plants develop tolerance towards soil salinity [9]. Rice (*Oryza sativa* L.) grown in salt-affected soil slightly impacted the K^+^ ion contents; however, it enhanced the Na^+^ contents in leaves and significantly lowered the K^+^/Na^+^ ratio [56,57]. Furthermore, a significant reduction was reported in the growth of strawberry plants [58]. These growth retardations could partially be attributed to reduced photosynthetic activity due to decreased *Chl a* and *Chl b* under various salinity levels [59]. The entrance of Na^+^ and Cl^−^ ions into the plant cell causes ion imbalance in plant and soil, and this ion imbalance in the plant might cause crucial physiological problems [60]. A high concentration of salts in the soil profile may cause physiological drought due to the reduction in water uptake and salt accumulation in the plant’s root zone [54], a decrease of plant osmotic potential, and thereby, the disturbance of cell metabolic functions due to ion toxicity [33,60]. Excess Na^+^ in plants harms the cell membrane and organelles of the plant, resulting in a reduction in plant physiological mechanisms such as the net photosynthesis rate (*Pn*), stomatal conductance (*Gs*), transpiration rate (*Tr*), intracellular carbon dioxide (*Ci*), and soil plant analysis development (SPAD) value, which lead to plant cell death [56,61,62]. In addition, these physiological changes in the plant might include the disruption of the cell membrane, leading to an inability to detoxify the reactive oxygen species (ROS) in the cytoplasm, a reduced photosynthetic rate and transformations of the antioxidant enzymes [62]. These oxidative systems can interrupt the routine functions of various plant cellular components such as proteins, DNA, and lipids, interfering with dynamic cellular functions in plants under abiotic stress, especially soil salinity [63]. Furthermore, plants grown in a saline environment might inhibit chlorophyll formation and trigger various modifications in the functions and structure of the pigment protein complex [64]. The inhibition of chlorophyll pigment synthesis under salt stress might be attributed to the declined activity of various enzymes, i.e., porphyrinogen IX oxidase, porphobilinogen deaminase, coproporphyrinogen III oxidase, 5-aminolevulinic acid dehydratase, protochlorophyllide oxidoreductase, and Mg chelatase [65]. Theses enzymes in turn are responsible for the upgradation of chlorophyllase activity [66] or a reduction in leaf water potential, N uptake, and thereby, the reduced photosynthetic capacity of plants [53]. Chlorophyll degradation might also be carried out by salinity-induced superoxide radicals and H_2_O_2_, which degrade the membranes of thylakoids and chloroplast [27].

### 2.3. Impacts of Salinity on Enzymatic and Non-Enzymatic Antioxidants

Soil salinity stress is accompanied with a robust accumulation of ROS and hampers plant growth and development. Under stressful circumstances (biotic and abiotic), reactive oxygen species (ROS): (O^2^, O^2−^, H_2_O_2_, and OH^−^) production is a stress indicator at a cellular level and is known as a secondary messenger which plays its role in the biological activities of plants, ranging from gene expression and translocation to enzymatic chemistry [67,68]. Ultimately, these ROS might cause alterations in the structures of lipids, proteins and nucleic acids, and thereby, cause an interruption of the normal plant metabolism [69]. It has been reported that soil salinity-stimulated oxidative stress due to the accretion of higher levels of H_2_O_2_ might induce apoptosis, cell shrinkage, chromatin condensation, and DNA fragmentation [70]. Under salinity stress, higher levels of ROS production might result in the production of malondialdehyde contents (MDA) in the thylakoid membranes. MDA concentration, which is known to be an effective indicator of lipid peroxidation, helps to calculate the lipid peroxidation of plant cells [71]. The balance between ROS production and their elimination by the antioxidative defense mechanism defines the degree of collateral damage to these molecules involved in plant metabolism [72]. Moreover, soil salinization causes acute oxidative damage in the plant tissues, and as a result, plants develop their own complex natural antioxidant defense system to combat with the salinity-induced oxidative stress [73]. The antioxidant enzymes inhibit the cell structural damages caused by salinity-induced ROS [74]. In the presence of an efficient antioxidant system in crop plants, it is believed that salt tolerance is better than for other types of plants. Previously, various researchers have reported the differential impacts of salinity stress on antioxidative enzymatic and non-enzymatic activities in *Tanacetum parthenium* L. [44], *Brassica napus* L. [47], *Oryza sativa* L. [75], and *Glycine max* L. [76]. The non-enzymatic antioxidative system mainly includes carotenoids, ascorbic acid (vitamin C), α-tocopherol, and flavonoids, while the enzymatic antioxidative system includes peroxidase (POD), superoxide dismutase (SOD), ascorbate peroxidase (APX), glutathione reductase (GR), polyphenol oxidase (PPO), etc. The major role of the enzymatic antioxidative system is to scavenge the injurious radicals produced during oxidative stress and thus help the crop plants to survive under abiotic stress such as soil salinity [67,77]. There are some natural antioxidants in almost all parts of the plant. These natural antioxidants are vitamins, carotenoids, phenols, dietary glutathione, flavonoids, and endogenous metabolites [78]. In salt-affected soils, the production and scavenging of these antioxidants makes up the first line of defense in plants to handle the oxidative stress.

## 3. Salinity Tolerance Mechanisms Adopted by Crop Plants

Plants have developed various adaptations at cellular, subcellular and organ levels for their nourishment under salt-affected soils. Some important salt resistance mechanisms are ion homeostasis, stomatal regulation, ion compartmentalization, osmoregulation/osmotic adjustment, hormonal balance changes, stimulation of the antioxidative defense mechanism, and the accumulation/exclusion of toxic ions from cells and tissues. However, all these salt-tolerant mechanisms are complex and vary from specie to specie [4]. According to biomass production under soil salinization, four plant groups are differentiated: (1) true halophytes (*Sued* sp. and *Atriplex* sp.), which can invigorate biomass production under salt stress; (2) optional halophytes (*Plantago maritima* and *Aster trripolium*), which show a minor increase in biomass at lower salt concentration; (3) nonresistant halophytes (*Hordeum* sp.), which can tolerate lower salt concentrations; and (4) glycophytes/halophytes (*Phaseolus vulgaris*), which are much more sensitive to salinization [79,80].

It has been suggested in many studies that salt tolerance is linked with the sequestration of Na^+^ ions into vacuoles after their entry into leaf cells to maintain a low Na^+^ concentration in the cytosol. This sodium and chloride ion compartmentalization phenomenon is carried out by proton gradient drove tonoplast Na^+^/H^+^ antiporters [46]. Once excess Na^+^ and Cl^−1^ are vacuolated, this significantly lowers the osmotic potential without any change in the metabolic process rate and ultimately contributes to osmoregulation [57]. Many experiments have emphasized this strategy, where the overexpression of vacuolar Na^+^/H^+^ antiporter gene (*NHX1*) family has enhanced the salinity tolerance of tomato (*Solanum lycopersicum* L.) [81], rice (*Oryza sativa* L.) [82], and maize (*Zea mays* L.) [29]. More recently, a novel virus-induced gene silencing (VIGS) method has been applied to study the function of GhBI-1 gene in cotton regarding the salt-stress response [83].

Excessive Na^+^ ion accumulation in plants is highly toxic because of its ability to interact with K^+^ ions, causing disturbed stomatal regulation. Therefore, the maintenance of a higher K^+^/Na^+^ ratio is an essential strategy for salt resistance in plants [2,6]. Two essential findings support this strategy: 1) the presence of *CED-9* gene enhanced salinity tolerance in tobacco by accumulating K^+^ ions [6] under salt-stressed conditions—more potassium is retained in the cell cytoplasm by caspase activity, i.e., proteases and endonucleases, [84]. Moreover, in salt-affected soils, the transfer of sodium and chloride ions in stem and leaf sheaths is another adaptation of crop plants to reduce the accumulation of these ions in more vulnerable leaf blades [85]. More precisely, it has been concluded that the K^+^/Na^+^ ratio in the cytosol can be retained by K^+^ absorption maintenance, the reduction of K^+^ efflux from cells, the prevention of Na^+^ uptake, and the enhancement of Na^+^ efflux from cells [86].

Generally, under stressful conditions, plant growth is also regulated by the synthesis of several phytohormones, such as jasmonic acid, salicylic acid, auxins, gibberellins and cytokinins (growth promoters) [87,88], ethylene, and abscisic acid (growth retardants). It has been reported that soil salinity enhanced the abscisic acid level in *Zea mays* L. at the expense of auxins (IAA) [89]. This modification may lead to the closing of stomata to reduce water loss as a consequence of osmotic stress under salinization. Methyl jasmonate, a natural plant growth regulator, can ameliorate the inhibitory effects of soil salinization on the photosynthetic rate to improve plant growth and development [90].

Another crucial physiological trait of salinity tolerance is the accumulation of organic compounds such as certain amino acids (proline, proline betaine, glycine betaine, and *β*-alanine betaine) and soluble sugars (fructose, glucose, fructans, raffinose, and trehalose). The accumulation of these compounds is positively correlated with salinity tolerance in *Zea mays* L. [91], *Pistacia vera* L. seedlings [46], and *Tanacetum parthenium* L. [44]. These compounds allow the maintenance of the turgor potential by decreasing the osmotic potential and minimizing the deleterious effects of Na^+^ ions against ribosomes and proteins. Recently, the exogenous application of different amino acids, proline, and glycine betaine was also considered as an ameliorative strategy for soil salinity [75,92,93,94].

A variety of adaptive mechanisms at the molecular level are involved in overcoming the harmful effects of salinity-induced oxidative stress. Some of the most important are the up and downregulation of gene transcripts [29,95,96], changes of chemical composition and the rigidness of plant’s cell wall [97]. It was reported that the expression of antioxidant defense genes is stimulated in *Zea mays* L. shoots [98], while Rodríguez-Kessler found that two genes, *Zmodc* and *Zmspds2A*, are responsible for salinity tolerance in maize roots through the accumulation of polyamine and spermidine [99].

## 4. Role of Selenium under Abiotic Stresses

Selenium (Se) has already been proven to be beneficial for humans and animals. However, Se is considered to be a double-edged sword due to its dual response to plants (beneficial or toxic) depending on its concentration and the nature of plant species [100]. Se is available in many forms to plants, such as selenate (Se, VI), selenite (IV), thioselenate, selenide, and elemental Se [101]. The optimum level of Se plays a crucial role in human and animal metabolism, e.g., a low concentration of Se in the diet is essential for antioxidant production and a healthy life and is recommended in many countries of the world. Thus, the effects of Se on humans and animals are linked with Se in the soil–plant system, because Se contents in edible parts of the plant come from the soil and are consumed by other organisms.

**Figure 2:**
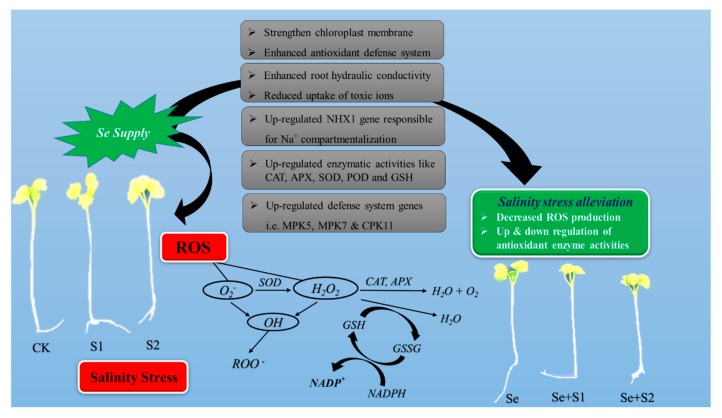
Schematic presentation showing the possible causes that overproduce reactive oxygen species (ROS), which might disturb the normal function of plant cells. The mechanism of antioxidants shown here scavenges the ROS effects as well as ameliorative effects of Se to induce salinity tolerance in crop plants. Se represents “selenium” (25 μM Na_2_SeO_4_) and S1 and S2 represent salinity stress (100 and 200 mM NaCl), respectively. The seedlings are representative of *Brassica napus* L. (Source: [24]). POD: peroxidase; SOD: superoxide dismutase; APX: ascorbate peroxidase; GSH: reduced glutathione; GSSG: oxidized glutathione; H_2_O_2_: hydrogen peroxide; NADP^+^: nicotinamide adenine dinucleotide phosphate; MPK: mitogen activated protein kinase gene; CPK: calcium dependent protein kinase gene; NHX: sodium/hydrogen (Na^+^/H^+^) exchanger gene.

Under low Se levels, it acts as an important protectant in plants grown under different abiotic and abiotic stresses. Selenium causes the disputation of ROS and protects plants from toxic elements-induced oxidative stress. At high levels, Se acts as a pro-oxidant as with other heavy metals/metalloids and enhances the production of ROS, causes protein oxidation, lipid peroxidation, and genotoxicity [102]. Selenium shows a hermetic effect in plants, but the mechanisms, as well as the optimal, essential, and toxic values of Se in the soil, are not well-established for different plant species and soil types. The essentiality of Se in plants depends on the plant species and Se concentration. For example, a hyper-accumulator species of *Brassica* species (*Helianthus*, *Camelina*, and *Aster*) could accumulate Se up to 100–1000 mg·kg^−1^ DW without showing toxicity symptoms. On the other hand, non-hyper-accumulator species of food crops, grasses, and vegetables hardly accumulate 100 mg·kg^−1^ DW of Se in plant tissues [103]. However, the Se response to salinity stress is not very clear and needs to be explored (Figure 2).

### 4.1. Selenium Speciation and Mobility in Soil

Selenium (Se) is present in excess in the Earth’s crust and can be beneficial or toxic to plants depending on the concentration of Se, speciation, and nature of plant species. Se occurs in organic and inorganic forms in soil with different oxidation states (+6, +4, 0, and −2) for selenate, selenite, elemental Se, and selenides, respectively. The most mobile and water-soluble inorganic Se is selenate (SeO_4_^2−^), which is present abundantly under oxic soil conditions with low adsorption affinity to oxide surfaces [104]. Selenate could be reduced into selenite due to poor adsorption ability onto the oxide surface under poor redox potential [102]. It has been demonstrated that selenite (SeO_3_^2^, HSeO_3_^−^, H_2_SeO_3_) might be the most abundant inorganic Se speciation under an anaerobic soil environment (pH: 7.5–15) [105]. At low pH, selenite has a greater ability to be adsorbed on an oxide surface than selenate and thus has reduced bioavailability to crop plants [104]. Selenite could be reduced into elemental Se, Se^0^, or selenides, Se^2−^ (unavailable to plants), under strong reducing conditions [102]. Various factors which are responsible for Se mobility and solubility in soil are soil pH, sorption, and desorption reactions, redox potential, organic/inorganic compounds, and dissolution processes in sediments and soils [106].

Soil Se is mainly inorganic but it can also be present in organic forms, such as complexes with organic matter, and incorporated into organic or organo-mineral colloids [107]. Se in organo-Se compounds (e.g., seleno-aminoacids) presents a valence state of −2 and is highly bioavailable. In addition, volatile organic forms of Se such as dimethyl selenide (DMSe) and dimethyl diselenide (DMDSe) may be present in soils. Se accumulation in plants is higher when seleno-amino acids are added to the hydroponic growth medium compared with inorganic forms of Se at the same concentration [108]. Organo-selenium compounds can either be released into the soil from biological decompositions of plant and soil microbial tissues or by Se-based fertilizer addition. Soil organic matter (OM) is shown to influence the retention of Se in [109]; however, the mechanisms of Se–OM interactions are poorly understood. Basically, three hypotheses explaining the OM-mediated retention of Se are generally discussed: (i) OM has increased sorption sites, which facilitates direct complexation with Se [109,110]; (ii) indirect complexation via OM–metal complexes [109]; (iii) microbial reduction and incorporation into amino acids, proteins, and natural organic matter [110]. Depending on the type of binding, Se may be easily mobilized (e.g., through pH adjustment) or immobilized (e.g., covalent incorporation to OM).

### 4.2. Selenium Uptake and Mobility within the Plants

The Se toxicity or deficiency margin is very small. This small gap between toxicity and essentiality is based on the nature of the organism and Se speciation [100]. It has been reported that a low-Se diet is important for antioxidant protection and a healthy life [111]. Therefore, threshold levels of Se have been added to the nutritional recommendation in various parts of the world such as China (essentiality: > 0.125 mg kg^−1^; toxicity: > 3 mg kg^−1^) [112]. Se deficiency or Se excess due to the intake of low or high-Se containing food may cause many health problems in living organisms [102]. Therefore, it is essential to understand and monitor the behavior of Se in the soil–plant system.

The majority of crop plants are able to uptake various inorganic forms such as selenite (+4), selenate (+6) [104], and/or various selenium based organic compounds such as SeCys (methylselenocysteine) and SeMet (selenomethionine) [105]. In contrast, plants are incapable to uptake elemental Se (0), selenide (−2) from the root zone. Even though Se is not an essential element for plants, it plays many significant roles in the plant, which depends on its applied concentration in the growth medium. Lower Se concentrations play a beneficial role and improve plant growth, whereas higher Se concentrations disturb the metabolic processes of the plant and reduce plant growth. The pathway of Se accumulation in plant roots is through specific and non-specific channels of essential nutrients (sulfur and phosphate), whereas the xylem channels and sinks transport *Se (VI)* into the shoot tissues within plants. Previously, it has been reported that phosphate transporter families (Pht1 and NIP2;1 transporter) are used to take up Se by root cells such as HSeO_3_^−^ and H_2_SeO_3_ (selenite) using aquaporins [113]. Afterwards, these Se speciations are translocated from root cells to the plant shoot as selenate via the root symplast and stele. During this whole process, selenite is persuaded into Se-based organic compounds, which stay behind in the plant roots [114,115]. Therefore, selenate and small amounts of SeMet and selenomethionine Se-oxide (SeOMet) have been considered important Se species in the plant xylem [116]. The family of aluminum-activated malate transporter (ALMT) genes are thought to be responsible for carrying selenate in the shoot xylem sap [117], whereas, following the delivery of selenate from root to shoot via the xylem, the members of the Sulfate transporters (SULTRs) family take it to leaf cells [118], where it is stored in the cell vacuoles [114].

In addition to inorganic Se, plant uptake of organic Se is known to occur and has been reported at much higher rates (20–100 fold greater) than the uptake of inorganic species [108]. Evidence suggests that amino acid transporters are important. To date, no Se-specific uptake mechanisms have been reported [119]. However, SeMet (selenomethionine), SeMeSeCys (Se-methyl selenocysteine) and SeCys (methylselenocysteine) forms of Se are taken up by the plant roots through transporters with the ability to catalyze the uptake of Met and Cys, respectively [120]. A synchrotron-based X-ray fluorescence microtomographic analysis was performed to demonstrate the transport mechanisms of organic species of Se. The authors observed that organic Se (SeMet and SeMeSeCys) was translocated in *Oryza sativa* L. exclusively via the phloem. The results indicated that, for SeMeSeCys- and SeMet-fed grain, Se was distributed throughout the external grain layers and into the endosperm, while SeMeSeCys Se was partitioned into the embryo. They demonstrated that organic Se species (SeMeSeCys and SeMet) are rapidly loaded into the phloem and transported to grain more efficiently than inorganic species [121].

## 5. Selenium-Mediated Alleviation of Salinity Stress in Plants

The findings to date have shown that Se is not ranked as an essential element for crop plants; however, a low Se concentration exerts beneficial effects on plant growth and development under biotic and abiotic stresses, especially soil salinization (Figure 2). Many studies have reported the effects of the application of Se to evoke tolerance against salt stress depending on the application method, dose of Se, salinity levels, and plant species [58]. For example, a foliar application of selenate (20 mg·L^−1^) mitigated the adverse effects of salinity stress (12 dS m^−1^) on the growth and development parameters of maize (*Zea mays* L.) [122]. Likewise, another study reported that Se application (20 µM) in the form of sodium selenite causes improvements in the growth and yield of eggplants under varying levels of soil salinity [123]. However, higher doses of selenite were found to have deleterious effects on the growth and development stages of maize under a salt stress of 100 mM NaCl [29]. Even though Se is an essential trace nutrient to humans and other animals as an antioxidant, Se toxicity might appear at higher concentrations due to the substitution of S with Se in the structure of amino acids, followed by the inaccurate folding of proteins and thus the creation of nonfunctional proteins and enzymes [102]. Conclusively, higher doses of Se hamper the growth and development of crop plants, while low doses cause improvements in growth and development mechanisms.

### 5.1. Improvement in Agronomic Traits

The maintenance of plant growth is directly associated with the survival of crop plants under salt-affected soils. The application of minute levels of Se under salinity stress significantly improved plant growth characteristics such as the shoot length, shoot diameter, and fresh and dry biomass of cucumber (*Cucumis sativus* L.), lemon balm (*Melissa officinalis* L.), cowpea (*Vigna unguiculata* L.), wheat (*Triticum aestivum* L.), and maize (*Zea mays* L.) as compared to salt stress alone [122,124,125,126] (Table 1). Likewise, Se showed a great potential to improve stem growth (diameter and biomass) in melon (*Cucumis melo* L.) and tomato (*Solanum lycopersicum* L.) when cultivated in salt-affected soils [30,106]. Recently, Astaneh suggested that growth parameters such as the bulb height, fresh and dry biomass of bulbs, bulb diameter, and the number of cloves in one bulb of Garlic (*Allium Sativum* L.) were significantly improved with the addition of Se under salinity stress [127]. Growth characteristics related to plant roots such as length, fresh, and dry weight were significantly improved with the supplementation of smaller amounts of Se alone and/or in combination with NaCl, compared to salinity stress alone [30,122]. Se applications significantly promoted root and shoot fresh weight and shoot dry weight as well as improving relative water contents in tomato (*Solanum lycopersicum* L.) and antioxidants activity and photosynthetic pigments in lettuce plants [100,128]. In addition, added Se also improved the growth parameters of ryegrass (*Lolium perenne* L.) and spinach (*Spinacia oleracea* L.) by improving nutritive values [106].

The accumulation of higher levels of Na^+^ ions in plant roots under salinity stress causes a reduction in hydraulic conductivity and ultimately lowered relative water contents (RWC); however, the Se (Na_2_SeO_4_) supply reduced Na^+^ ions and improved root growth, and thereby, might have enhanced the water supply to shoots and sustained plant growth [27,139]. Salt-affected soils cause hindrances in nitrogen assimilation, accumulation, and metabolism, and hence, disturb the proline (a molecular chaperone responsible for maintaining protein integrity) biosynthetic mechanism [140,141]. The improvement in the phenological parameters of crop plants could also be a consequence of Se-mediated increments in proline contents through the promotion of nitrogen (N) contents and nitrate reductase activity [53]. Furthermore, Se has been involved in the improvement of nutrient elements absorption and their transfer within the body of various crop plants, which ultimately improves growth and production [142]. It was stated that suitable Se supplementation might be involved in boosting the expression of tonoplast H^+^ ATPase and Na^+^/H^+^ antiport at the root membranes, limiting Na^+^ ion translocation to the upper plant tissues, thus, decreasing its toxic impacts [143]. Moreover, cations such as nitrogen, potassium, and calcium are required for growth regulation through their impact on the vital metabolic pathways such as antioxidant metabolism, nitrogen assimilation, and cellular stress signaling [72,91,144]. The Se supply has been reported to be beneficial to increasing the nitrogen, potassium, and calcium uptake from soils, thereby, leading to a larger production of amino acids, metabolites, and stress signaling for better induction of salinity tolerance in wheat (*Triticum aestivum* L.) [28]. Another important mechanism is Se-accelerated reduction in the Na^+^/K^+^ ratio in plants grown in salt-affected soils, which ultimately induces the protection of some essential processes and balanced osmotic potential [127]. Na^+^ ions are responsible for inhibiting K^+^ ion uptake at the membrane transport level, whereas Se might have the ability to influence the expression of Na^+^ transporters and H^+^ pumps [145].

### 5.2. Se-Mediated Improvement in Physiological Attributes

To situate the scientific context compiled in this review article, it should be taken into account that Se at low concentrations helped plants to alleviate exposed stress from its exterior environment, especially regarding soil salinity. Therefore, an exogenous application of Se has gained considerable interest in the scientific community around the world [22,24,94]. For instance, exogenously applied Se played a significant role in appraising the physiological and biochemical mechanisms (Table 1) involved in salinity tolerance in cucumber [124], canola [24], and parsley [133], which as a result helped plants to survive better in salt-stressed environments. Salinity stress in particular not only damages a plant’s osmotic potential, but also accompanies various secondary stresses, such as cellular oxidative damage by the over-generation of reactive oxygen species (ROS) [122]. The maintenance of ROS homeostasis and other physiological functions such as photosynthesis are the chief priorities of plants exposed to salinity stress [29]. Therefore, finding suitable approaches to understand and investigate the mechanisms underpinning plant responses to salinity stress is essential to sustain agricultural production in saline soils. In this regard, the application of Se has been found to reduce the harmful effects of salinity and support the growth of maize (*Zea mays* L.), tomato (*Solanum lycopersicum* L.), and garlic (*Allium sativum* L.) through enhanced photosynthetic performance [29,30,122,129]. Moreover, enhanced growth and nutritional qualities of spinach (*Spinacia oleracea* L.), ryegrass (*Lolium perenne* L.), wheat (*Triticum aestivum* L.), and mung bean (*Vigna radiate* L.) have also been reported by exogenously applied Se under stressed and non-stressed conditions [22,106,142,146]. Further, a lower Na^+^ concentration and higher K^+^/Na^+^ ratio was observed in selenite-treated plants as compared to untreated plants [27]. Se might have decreased the accretion of Na^+^ ions which led to an increased K^+^/Na^+^ ratio in comparison to the untreated control plants of dill (*Anethum graveolens*) and garlic (*Allium sativum* L.) [129,147]. The addition of Se under salinity stress significantly improved the physico-biochemical properties such as the chlorophyll contents, carbohydrates, proteins, and carotenoids, of which adequate amounts are essential to regulate major metabolic processes such as photosynthesis in maize (*Zea mays* L.) [148]. The application of Se significantly improved the plant growth, photosynthetic activities such as the net photosynthetic rate, the actual photochemical efficiency of photosystem II (PSII), maximum quantum yield of PSII (*Fv*/*Fm*), photochemical quenching coefficient (*qP*), and non-photochemical quenching coefficient (*qN*) of tomato (*Solanum lycopersicum* L.) cultivars [30]. Similarly, Se application showed a positive effect on growth and improved the photosynthetic pigments and total amino acid contents in lemon balm (*Melissa officinalis* L.) and decreased Na^+^, while increasing K^+^ concentrations in the roots and shoots of dill (*Anethum graveolens*) plants [133,147]. Furthermore, many other researchers have shown that Se application to salt-stressed cucumber and tomato protected the cell membranes against lipid peroxidation, reduced oxidative stress by regulating the chloroplast, which is strongly linked with increasing the photosynthetic rates by improving the PSII, and thereby, enhanced plant stability [30,124]. Taken together, these findings suggest that Se played a significant role in improving the physiological and biochemical adaptation of plants, which eventually helped plants to survive better in stressed saline conditions.

It has been recognized previously that the amelioration of photosynthetic inhibition through Se supply might be a result of the cumulative impact on the antioxidative defense mechanisms, leading to the simultaneous alleviation of ROS effects, uptake and accumulation of important crop nutrients [149]. Recently, it was shown that a higher Se supply (10 µM) causes retardation in the growth and photosynthetic capacity of wheat (*Triticum aestivum* L.) seedlings [28], which might be attributed to decreased chlorophyll formation due to the inhibition of chlorophyll biosynthesizing enzymes and production of 5-aminolevulinic acid and protochlorophyllide [150]. An increment in Mn, Zn, and Fe contents in plant leaves under Se treatment [151] could also be the reason for the improved photosynthetic apparatus and avoidance of the degradation of chlorophyll [152]. Optimal supplementation of Se modulates photosynthetic functioning by enhancing CO_2_ assimilation, photosynthetic rate, and chlorophyll fluorescence characteristics under normal and stressful conditions [149]. Moreover, a Se supply regulated proline accumulation by enhancing the activity of γ-glutamyl kinase (γ-GK) enzyme, leading to the enhanced synthesis of proline with subsequent declines in its degradation via the slowing down of the activity of proline oxidase [28,153]. In halophytic grasses, it has been demonstrated that increased accumulation of proline leads to enhanced photosynthetic efficiency and ATP production, resulting in greater water use efficiency [154]. The above discussion reveals that the application of a low concentration of Se could play an important role in the improvement of the physiological and defensive mechanisms of crop plants under salinity stress.

### 5.3. Se-Mediated Improvement in the Alleviation of ROS Effects

Plants produce an array of antioxidant enzymes once exposed to biotic and abiotic stresses and, interestingly, Se supplementation has been found to upscale these antioxidant enzyme activities to cope with experienced stresses [155]. Se has a significant role in numerous enzymatic processes—i.e., catalase (CAT), peroxidase (POD), superoxide dismutase (SOD), ascorbate peroxidase (APX), and glutathione peroxidase (GPX)—and non-enzymatic processes—i.e., phytochelatins and glutathione antioxidants—which help to combat the salt-induced overproduction of reactive oxygen species (ROS), which are responsible for agitating plant cell integrity (Figure 2). Molecular oxygen (O_2_) works as an electron acceptor with a subsequent accretion of reactive oxygen species (ROS) such as singlet oxygen (^1^O_2_), hydroxyl radical (OH^−^), superoxide radical (O^−2^), and hydrogen peroxide (H_2_O_2_) under salt-stressed conditions. It has been well proven that lower concentrations of selenate (Na_2_SeO_4_) help to protect plants from ROS-stimulated oxidative damage, but a higher concentration of Se works as a pro-oxidant and stimulates the formation of ROS and induces oxidative stress [92]. Many researchers have described that Se is required to increase the scavenging activity of ROS, decreasing the concentration of MDA and membrane damage [156]. Moreover, decreased generation of H_2_O_2_ under Se supplementation has also been confirmed [157,158]. Under salinity stress, lowered H_2_O_2_ contents were observed in Se-treated canola (*Brassica napus* L.) plants [136]. Meanwhile, plants exposed to Se showed lower concentrations of MDA under NaCl stress, showing that Se was vital in bringing down the lipid peroxidation by amending the antioxidant enzymes and protecting the membranous structures of *Oryza sativa* L. [27], *Cucumis sativus* L. [124], *Brassica napus* L. [24], and *Anethum graveolens* [147]. In addition, it was noticed that lipid peroxidation (MDA) production was reduced by elevating Se concentration under salt stress [127]. A comprehensive impact of MDA on plant cells is lowering the fluidity of the membranes to elevate membrane leakiness and avoiding damage to membrane proteins, enzymes, and ion channels [159]. A suitable concentration of Se might be useful to limit the over-expression of lipidoxygenease for sustaining fatty acid formation in addition to the lessened ROS generation, which was led by the upregulation of antioxidant systems [28].

### 5.4. Se-Mediated Improvement in the Upregulation of Enzymatic and Non-Enzymatic Antioxidants

Under soil salinity stress, ROS can be detoxicated by antioxidant compounds (Figure 2; Table 2). It is believed that enzymatic and non-enzymatic antioxidants, such as SOD, POD, APX, CAT, GSH-Px, and GR, are positively interconnected in response to Se supplementation to induce salinity tolerance in crop plants [22,131]. Researchers postulate that an elevation in the Se-mediated antioxidant defense is one of the vital mechanisms that can save plants from salt-stimulated oxidative stress [58,134]. Antioxidant enzyme activities (SOD, APX, and CAT) significantly improved with exogenous Se treatment in rapeseed (*Brassica napus* L.) and dill (*Anethum graveolens*) seedlings under salinity stress [24,147]. In another study, the accumulation of lowered H_2_O_2_ contents in rice plants might have been due to Se-mediated higher levels of APX and CAT activities [27]. An increment in the activities of SOD, CAT, GST, APX, and GR has been noticed in different crops such as *Triticum aestivum* L., *Brassica juncea* L., *Avena sativa* L., and *Solanum lycopersicum* L. [13,144,160,161]. Recently, it was noticed that the translocation of minerals such as iron, zinc, and manganese was significantly increased in the shoots of rice (*Oryza sativa* L.) with Se application [151]. These minerals are essential components of antioxidant enzymes and responsible for increasing the activities of SOD, POD and CAT [162]. Under salinity stress, the exogenous supplementation of Se to maize (*Zea maize* L.) plants resulted in the upregulation of expression of mitogen activated protein kinase (*MAPK*5 and *MAPK*7) and calcium-dependent protein kinase (*CPK*11) genes and stimulated the antioxidant defense system under salt stress [29,163]. It has been reported that *MAPK* flow is at the center of cell signal transduction and implicated in stress-related signal pathways [164]. Abscisic acid (ABA) accumulation could be stimulated under salinity stress [165], which in turn produces H_2_O_2_, causing the activation of *MAPK*, resulting in stimulated expression and activities of antioxidant enzymes [166]. Furthermore, NAD kinase-2 (NADK2) mutation impaired ABA-induced stomatal closure and ABA inhibition of light-promoted stomatal opening. NADK2 disruption also impaired the ABA-stimulated accumulation of H_2_O_2_ [167,168]. Elevation of SOD activity due to Se supplementation evolved in the quick transformation of the superoxide radicals into H_2_O_2_, which was produced at the chloroplast and mitochondrial electron transport chain. The evolving H_2_O_2_ was counteracted either by CAT in the cytoplasm or by APX in the ascorbate glutathione (AsA–GSH) pathway. Furthermore, increased SOD activity in Se-supplemented seedlings altered the chances of hydroxyl (OH^−^) radical composition, following a better defense of chloroplast function [162].

The scavenging of H_2_O_2_ and lipid peroxide (MDA) into water and lipid alcohol is done by two important enzymes: glutathione peroxidase (GSH-Px) and glutathione reductase (GR) [20]. GSH-Px is considered to be a vital enzyme, which is strongly activated by Se in different plants under various environmental stresses [173]. In the presence of Se, GSH-Px quenches H_2_O_2_ and then APX, CAT, and GR remove the leftover of H_2_O_2_. Under salinity stress, regardless of the mode of Se application, Se enhanced the GSH-Px and GR activity compared to controls [27,28]. Under the availability of Se, GSH-Px activity might be modulated due to higher selenocysteine formation at the catalytic site of GSH-Px [27,173]. The enhanced activity of GSH-Px and GR lowered the levels of H_2_O_2_ and MDA and improved the growth of rapeseed (*Brassica napus* L.) and rice (*Oryza sativa* L.) plants by overcoming ROS-stimulated oxidative damage under soil salinity stress [24]. APX lowers the level of H_2_O_2_, while GR impacts the preservation of GSH and AsA content resulting in reasonable cellular redox [72]. The supplementation of Se in wheat (*Triticum aestivum* L.) seedlings upregulated the AsA–GSH pathway by increasing the activities of APX and GR. Furthermore, elevating the AsA and GSH contents consistently evolved in the defense of the photosynthetic electron transport chain by sustaining better nicotinamide adenine dinucleotide phosphate (NADP^+^) levels and limiting the composition of toxic radicals [28]. These results revealed that the wise use of Se could be beneficial to improving the plant antioxidative defense mechanism under soil salinity stress.

### 5.5. Se-Mediated Gene Expression Modifications under Salinity Stress

Very few studies have elucidated the role of Se in the alleviation of Na^+^ accumulation and its hazardous impacts on plant growth and development at the gene level. In an experiment on maize (*Zea mays* L.), Jiang investigated the expression levels of associated genes such as *ZmMPK5*, *ZmMPK7*, and *ZmCPK11*, which are responsible for the antioxidant defense system in roots, while the expression of *ZmNHX1* gene clarified Se’s involvement in Na^+^ and K^+^ homeostasis under salt-affected soils [29] (Figure 2). In previous studies, the contribution of genes to the removal of ROS has been well documented. It has been reported that H_2_O_2_ is the activator of *ZmMPK5*, and hence, the antioxidant defense system of maize leaves was enhanced [174]. Similarly, the expression of *ZmCPK11* increased the activities of APX and SOD in maize (*Zea mays* L.) leaves [175]. Moreover, under a stress salt environment, the *ZmMPK7* gene was found to be a good alleviator of ROS-induced damages in tobacco (Nicotonia tobaccum L.), resulting in low H_2_O_2_ accumulation [155]. Likewise, it was described that a small amount (1 µM) of Se (Na_2_SeO_3_) addition under osmotic stress enhanced the upregulation of *ZmMPK5*, *ZmMPK7*, and *ZmCPK11* genes in roots of maize (*Zea mays* L.) [29]. In many previous findings, *NHX* gene overexpression in transgenic plant species—i.e., rapeseed [176], tomato [81], and poplar [14]—is responsible for Na^+^ compartmentalization and an enhancement of salt resistance. Recently, it was proven that *ZmNHX1* expression was significantly up-regulated in maize after 24 h of salinity stress exposure, which may contribute to Na^+^ compartmentalization under osmotic stress [29].

Furthermore, it was reported that *OsNHX1* (vacuolar Na^+^/H^+^ antiporter gene) is responsible for maintaining plant osmotic balance by reducing the hindrance of Na^+^ ions during water movement towards plant shoots [14], which might be due to the sequestration of sodium ions in vacuoles of roots and/or shoots [177]. Previously, this phenomenal mechanism was strengthened by research work on tomato (*Solanum lycopersicum* L.) and rapeseed (*Brassica napus* L.), respectively [81,176]. Recently, Se (Na_2_SeO_4_) was supplied to salinity-stressed rice (*Oryza sativa* L.) plants grown under a saline environment in a mixture of sand and polymer, and it was observed that plants receiving Se exhibited a higher transcription level of *OsNHX1* gene [27]. The researchers concluded that it could be imagined that a higher *OsNHX1* transcript level promoted Na^+^ sequestration within the root vacuoles and therefore reduced the Na^+^ accumulation in rice shoots, which ultimately improved plant growth and antioxidative defense mechanism. However, further research work is needed to explore how Se is involved in antioxidant defense genes and how these genes are up and downregulated to induce antioxidant defense systems in salt-stressed plants under Se supplementation.

## 6. Conclusions and Future Perspectives

Soil salinization has become an overwhelming environmental threat to world food production and agricultural sustainability. Selenium (Se) is recognized as an essential trace element for human beings and animals, although this is controversial for different plant species. However, based on published relevant literature, it is widely accepted that Se is capable of remediating various biotic and abiotic environmental stresses including soil salinity. The important mechanisms involved in Se-mediated salinity tolerance in crop plants include a reduction in Na^+^ ion accumulation in plant parts through the overexpression of the Na^+^/H^+^ antiport, chelation and boosting of the antioxidative defense system in plants, Na^+^ compartmentalization, improvement in various structural compositions, and the upregulation of Na^+^ and Cl^−^ ions transporter genes. However, these salinity-tolerance mechanisms are still highly controversial and are influenced by growth conditions, growth medium (soil or water), stress duration, plant genotypes, plant species types, Se doses, speciation, and many more. Therefore, it is difficult to predict a general conclusion for the Se-mediated alleviation of salinity-induced phytotoxicity in crop plants. More precisely, at lower concentrations, Se can mediate plant growth and physiological characteristics (acts as a beneficial element), while at higher concentrations; it disturbs various plant metabolic processes, and thereby suppresses plant growth under salinity stress. Moreover, Se triggers the dismutation of ROS generated under salt stress and protects plants from oxidative damage. In conclusion, this review article has shed light on the hazardous impacts of soil-affected soils, various salinity tolerance mechanisms adopted by crops and the prospective mechanisms involved in Se-mediated salinity tolerance as well as improvements in the growth and productivity of various crop plants cultivated in salt-affected soils.

In this review paper, after critically reviewing the best available data to date, the authors anticipated that there would be an emergent interest in the scientific community to studying the mechanisms of Se-assisted salinity tolerance in plants in the near future; therefore, the following research gaps need to be explored in future.

On an instructive note, the suitable concentration of Se supplementation is still a matter of research. Complete interpretation of the role of Se as well as detailed protective mechanisms would be helpful for developing salinity tolerance in plants.

The Se transformations in the plants are still unclear. Therefore, future studies are required to explore the exact mechanisms involved in Se transformations inside plant species that enhance Se transfer to the plant shoots and its volatilization from aerial plant parts.

Previous researchers have focused on evaluating the role of Se in individual plant species grown in salt-affected soils; however, there is still a need to better understand its ameliorative roles in more plant species under various environmental factors for the confirmation of the Se-mediated amelioration of salinity-induced phytotoxicity on a larger scale.

According to the reviewed data, Se in most experiments was used under saline nutrient mediums (hydroponics). Such experimental results can overestimate the Na^+^ uptake and translocation within the plant body. It is advised to conduct future experiments on natural saline soils (pots or field), as soil is a complex system, which will provide a better understanding of Se-mediated salinity-tolerance mechanisms. Moreover, such experiments will help the local farming community to learn about the use of Se in farming practices.

More importantly, to date, most soil-based experiments have been executed over the short term, which raises questions on Se’s potential to remediate salt-affected soils in the long term. Therefore, well-planned, comprehensive, and long-term field experiments are needed to check the productivity and economic feasibility of Se-based ameliorations of saline soils.

Despite the widespread occurrence of Se deficiency globally, Se toxicity (selenosis) is a problem in some areas. Some soils and mineral deposits are naturally Se rich, and exploitation of these seleniferous soils can lead to toxic accumulation of Se in the environment. Therefore, effective enrichment of agricultural crops with Se via soil using Se-enriched fertilizers can be challenging due to varying soil Se concentrations, soil types, soil redox potentials, soil pH, and microbiological activity. Furthermore, the high cost of Se fertilizer, in combination with the modest incorporation rate, should be considered.

## Figures and Tables

**Table 1:** Protective effects and mechanisms of Se supplementation on growth, physiological, and biochemical attributes of plants grown under salinity stress.

Salinity Stress	Plant Species	Se Dosages	Se Speciation	Experimental Details	Various Protective Effects and Mechanisms of Se in Salinity Stressed Plants	References
150 mM	*Oryza sativa* L.	2, 4, 6, 8, 10, 12 mg·L^−1^	*Se (VI)*	Sand culture	Enhances plant biomass, K^+^/Na^+^ ratio, and Se accumulation; reduces malondialdehyde contents (MDA) and H_2_O_2_ contents; increases chlorophyll and water contents; causes upregulation of *OsNHX1* gene transcript levels	[27]
0, 30, 60, 90 mM	*Allium sativum* L.	0, 4, 8, 16 mg·L^−1^	*Se (VI)*	Hydroponic culture	Increases root biomass, bulb diameter, bulb height, and photosynthetic pigments; reduces ion leakage and lipid peroxidation; improves K^+^ and Na^+^ contents, chlorophyll index, carotenoids, and water balance	[127,129]
100 mM	*Triticum aestivum* L.	5, 10 µM	*Se (VI)*	Reconstituted soil culture (Peat, compost, sand)	Improves wheat growth; promotes the synthesis of photosynthetic pigments, proline, and sugars; reduces H_2_O_2_ contents, Na^+^ uptake, and Na^+^/K^+^ ratio	[28]
10, 30, 60, 90 mM	*Stevia rebaudiana* Bertoni	20 g/ha (2 ppt)	*Se (IV)*	Field experiment	Increases leaf and plant biological yields; enhances rebaudioside-A and stevioside of stevia leaves; improved the accumulation of sweet steviol glycosides contents	[21]
0.12, 0.30, 0.60 S m^−1^	*Triticum aestivum* L.	0, 0.5, 1, 4 mg·kg^−1^	*Se (IV)*	Pot soil culture	Dramatic decrease in shoot dry biomass; chlorophyll a, chlorophyll b, and carotenoid contents increase at lower Se, while they decrease at higher Se; enhances free proline and Se contents in shoots;	[130]
8 dS m^−1^	*Allium cepa* L.	0, 0.5, 1 kg·ha^−1^	*Se (IV)*	Field experiment	Increases bulb yield and dry matter; improves water and chlorophyll contents; causes bulb Se and K enrichment; causes a decrease in Na	[131]
0, 100 mM	*Phaseolus vulgaris* L.	0, 5, 10 μM	*Se (IV)*	Pot soil culture	Enhances plant growth and seed yield; promotes membrane stability index, photosynthetic capacity, and RuBPCase activity; reduces (MDA) and electrolyte leakage	[132]
0, 100 mM	*Zea mays* L.	0, 1, 5, 25 μM	*Se (IV)*	Pot vermiculite culture	Enhances growth and biomass; improves gas exchange attributes and the shape of thylakoids by alleviation of damage in the ultrastructure of chloroplasts; upregulates *ZmMPK5*, *ZmMPK7*, *ZmCPK11*, and *ZmNHX1* genes transcript levels in roots	[29]
0, 80 mM	*Petroselinum crispum* L.	1 mg·L^−1^	*Se (VI)*	Hydroponic culture	Decreases root to shoot transport of Na^+^; improves photochemical efficiency of photosystem II (PSII) and chlorophyll contents; protects photosynthetic apparatus by upregulation of non-photochemical quenching (*NPQ*); decreases cell sap Na^+^	[133]
3.22 dS m^−1^	*Lactuca sativa* L.	16, 32 µM	*Se (VI)*	Field experiment	Improves growth characteristics, yield, and relative water contents; decreases cell membrane permeability and malondialdehyde; enhances chlorophyll, carotenoids, K^+^/Na^+^, and total soluble sugars	[134]
0, 25, 50 mM	*Lycopersicon esculentum*-Mill.	0, 5, 10 µM	*Se (IV)*	Hydroponic culture	Enhances growth by improving water balance and cell membrane integrity; increases photosynthetic pigments; decreases proline and phenolics	[128]
0, 30, 60, 120 mM	*Solanum melongena* L. cv. Baladi	0, 5, 10, 20, 30 µM	*Se (IV)*	Bedding sand culture	Increases vegetative growth, yield, nitrogen, phosphorous and potassium NPK contents in leaves and fruits; improves chlorophyll contents (SPAD value) and proline contents; Enhances K^+^/Na^+^ ratio	[135]
0, 40 mM	*Lactuca sativa* L. var. capitate	0, 2, 6 µM	*Se (IV, VI)*	Hydroponic culture	Enhances fresh biomass, leaf area, chlorophyll, proline, and carotenoid contents; reduces H_2_O_2_ and *TBARS*; improves shoot ionic concentrations	[100]
0, 40 mM	*Melissa officinalis* L.	10 mM	*-*	Hydroponic culture	Improves growth rate; increases photosynthetic pigments, protein, and total amino acid contents; reduces lipid peroxidation to alleviate membrane damage	[125]
0, 2000, 4000, 6000 mg L^−1^	*Brassica napus* L.	0, 2.5, 5, 10 mg·L^−1^	*Se (VI)*	Pot clay soil culture	Enhances growth, photosynthetic pigments, canola oil quality; increases soluble sugar, polysaccharides, and total carbohydrates; significantly improves saturated and unsaturated fatty acids composition	[136]
0, 2000 ppm	*Cucumis sativus* L. *cv* Zena	0, 1 ppm	*Se (IV)*	Pot soil culture	Improves plant biomass; increases reduction of oxygen radicals and osmotic regulation by synthesis of osmoregulatory compound such as proline; reduces malondialdehyde concentration and electrolyte leakage	[137]
0, 50 mM	*Cucumis sativus* L.	0, 5, 10, 20 µM	*Se (VI)*	Hydroponic culture	Induces salt tolerance by protection of cell membranes against lipid peroxidation; improves growth rate, photosynthesis, and proline contents; reduces Cl^−^ contents, while showing no effect on Na^+^ ions and K^+^/Na^+^ ratio	[124]
100 mM	*Rumex patientia × R. tianshanicus*	0, 1, 3, 5, 10, 30 µM	*Se (IV)*	Sand culture	Increases seedling growth; lower Se supply improves total water-soluble sugars, K^+^, and Na^+^ concentrations; alleviates integrity of cytoplasmic organelles, plasma and nuclear membranes, root tip cells; makes more legible and increases mitochondrial cristae in leaf mesophyll	[138]

The abbreviations are explained in the list of abbreviations.

**Table 2:** Selenium (Se) supplementation mitigates salinity-induced oxidative damage by changing different antioxidant enzymatic and non-enzymatic activities in the leaves of different salt-stressed plants (↑ indicates an increase, while ↓ indicates a decrease).

Salinity Stress	Plant Species	Se Dosages	Se Speciation	Experimental Details	↑↓ Antioxidant Activity	% Increase or Decrease	Reference
150 mM	*Oryza sativa* L.	2, 4, 6, 8, 10, 12 mg·L^−1^	*Se (VI)*	Sand culture	↑SOD ↑APX ↑CAT ↑GR ↑GSH-Px	40.7% 92.7% 82.9% 77.2% 66.1%	[27]
0, 25, 50, 75 mM	*Fragaria* × *ananassa* Duch	0, 10, 20 mg·L^−1^	Se-NPs	Reconstituted pot culture (perlite, peat, sand)	↑SOD ↑POD	35.9% 63.1%	[58]
100 mM	*Triticum aestivum* L.	5, 10 µM	*Se (VI)*	Reconstituted pot culture (Peat, compost, sand)	↑SOD ↑CAT ↑GST ↑APX ↑GR	16.2% 10.1% 16.2% 10.6% 22.1%	[28]
0, 30, 60, 90 mM	*Allium sativum* L.	0, 4, 8, 16 mg·L^−1^	*Se (VI)*	Hydroponic culture	↑SOD ↑CAT ↓POX ↑PAL	81.0% minute minute ~15.0%	[127,129]
12 dS m^−1^	*Zea mays* L.	0, 20, 40 mg·L^−1^	*Se (VI)*	Sand culture	↑CAT ↑POD ↑SOD	~56.0% ~63.0% minute	[122]
0, 100 mM	*Phaseolus vulgaris* L.	0, 5, 10 μM	*Se (IV)*	Pot soil culture	↑SOD ↑POD ↑CAT	15.8% 313.3% 56.3%	[132]
8 dS m^−1^	*Allium cepa* L.	0, 0.5, 1 kg·ha^−1^	*Se (IV)*	Field experiment	↓CAT ↓POD	26.6% 10.0%	[131]
0, 25, 50 mM	*Lycopersicon esculentum*-Mill.	0, 5, 10 µM	*Se (IV)*	Hydroponic culture	↓POD ↑CAT	60.0% ~240.0%	[128]
0, 50 mM	Vigna unguiculata L.	5, 10 µM	*Se (VI)*	Sand-soil culture	↑SOD ↑POD ↑PAL	63.4% 238.1% 73.5%	[169]
0, 100 mM	*Vigna radiata* L. Wilczek	1, 2.5, 5 ppm	*Se (VI)*	Reconstituted pot culture(Soil, sand, farmyard manure)	↑SOD ↑CAT ↑APX ↑GR ↑GPX	14.2% 37.0% 34.8% 24.6% 41.0%	[170]
0, 10 dS m^−1^	*Anethum graveolens* L.	0, 5 µM	*Se (VI)*	Hydroponic culture	↑CAT ↑SOD ↓APX	~40.0% ~19.0% minute	[147]
0, 100 mM	*Lycopersicon esculentum-Mill.* Shuangfeng 87-5	0.05 mM	*Se (IV)*	Hydroponic culture	↑GR ↓APX ↑DHAR ↑MDAR	~23.0% ~14.0% ~50.0% ~16.0%	[30]
0, 100 mM	*Glycine max* var. L17	0, 25, 50 mg·L^−1^		Pot soil culture	↑CAT ↑POD ↑SOD	221.6% 85.0% 40.0%	[171]
0, 100 mM	*Cucumis melo* L.	0, 2, 4, 8, 16 μM	*Se (IV)*	Hydroponic culture	↑POD CAT ↑SOD	~29.0% unchanged ~106.0%	[172]
0, 100, 200 mM	*Brassica napus* L.	25 µM	*Se (VI)*	Semi-hydroponic culture	↑GSH ↑GSH/GSSG ↑DHAR ↑MDHAR ↑GST ↑GR	33.0% 86.0% 43.0% 45.0% 18.0% 40.0%	[24]

The values of % increase or decrease in antioxidant activities represent the NaCl and Se treatment dosages mentioned in bold characters. “~” indicates approximate values.

## Abbreviations

Se-NPs	Selenium-nanoparticles
GSH	Reduced glutathione
GSSG	Oxidized glutathione
DHAR	Dehydroascorbate reductase
MDHAR	Monodehydroascorbate reductase
GST	Glutathione S-transferase
GR	Glutathione reductase
POX	peroxidase
PAL	Activity of phenylalanine ammonia-lyase
GSH-Px	Glutathione peroxidase
CAT	Catalase activity
APX	Ascorbate peroxidase activity
SOD	Superoxide dismutase activity
POD	Peroxidase activity
GPX	Glutathione peroxidase activity
MDAR	Monodehydroascorbate reductase activity
RWC	Relative water contents
TBARS	Thiobarbituric acid reactive substances
NPQ	Non-photochemical quenching
MDA	Malondialdehyde
RuBPCase	Ribulose-1,5-bisphosphate-carboxylase/oxygenase content
SPAD	Chlorophyll content in leaves
H_2_O_2_	Hydrogen peroxide
ATP	Adenosine triphosphate
NADP^+^	Nicotinamide adenine dinucleotide phosphate
*MAPK*	Mitogen activated protein kinase gene
*CPK*	Calcium dependent protein kinase gen
NADK2	NAD kinase2 gene
ALMT	Aluminum-activated malate transporters
SULTRs	Sulfate transporters
γ-GK	γ-Glutamyl kinase
NHX	Sodium/hydrogen (Na^+^/H^+^) exchanger gene
PSII	Photosystem II
NPK	Nitrogen, phosphorous, and potassium
